# Scalable lateral heterojunction by chemical doping of 2D TMD thin films

**DOI:** 10.1038/s41598-020-70127-6

**Published:** 2020-07-31

**Authors:** Bhim Chamlagain, Sajeevi S. Withanage, Ammon C. Johnston, Saiful I. Khondaker

**Affiliations:** 1 Department of Physics, NanoScience Technology Center, University of Central Florida, Orlando, FL 32826 USA; 2 School of Electrical Engineering and Computer Science, University of Central Florida, Orlando, FL 32826 USA

**Keywords:** Nanoscience and technology, Nanoscale devices, Nanoscale materials

## Abstract

Scalable heterojunctions based on two-dimensional transitional metal dichalcogenides are of great importance for their applications in the next generation of electronic and optoelectronic devices. However, reliable techniques for the fabrication of such heterojunctions are still at its infancy. Here we demonstrate a simple technique for the scalable fabrication of lateral heterojunctions via selective chemical doping of TMD thin films. We demonstrate that the resistance of large area MoS_2_ and MoSe_2_ thin film, prepared via low pressure chalcogenation of molybdenum film, decreases by up to two orders of magnitude upon doping using benzyl viologen (BV) molecule. X-ray photoelectron spectroscopy (XPS) measurements confirms n-doping of the films by BV molecules. Since thin films of MoS_2_ and MoSe_2_ are typically more resistive than their exfoliated and co-evaporation based CVD counterparts, the decrease in resistance by BV doping represents a significant step in the utilization of these samples in electronic devices. Using selective BV doping, we simultaneously fabricated many lateral heterojunctions in 1 cm^2^ MoS_2_ and 1 cm^2^ MoSe_2_ films. The electrical transport measurements performed across the heterojunctions exhibit current rectification behavior due to a band offset created between the doped and undoped regions of the material. Almost 84% of the fabricated devices showed rectification behavior demonstrating the scalability of this technique.

## Introduction

Two dimensional (2D) layered materials are considered to be promising candidates for applications in electronics, optoelectronics, catalysis, sensor, and energy storage devices^1–6^. In particular, heterojunctions of 2D van der Waals materials involving graphene and transition metal dichalcogendies (TMDs) semiconductors have created enormous attention due to their potential opportunities of designing novel device structures for the discovery of novel physics as well as for their potential applications in the next generation of electronic and optoelectronic devices^6–17^. However, the majority of the techniques that has been demonstrated for heterojunction fabrications are complex and suffer from low yield making them not suitable for scalable device fabrication. Van der Waals heterojunction was first demonstrated in a graphene—tungsten disulfide (WS_2_) system and was fabricated by stacking graphene and mechanically exfoliated flakes of WS_2_ via deterministic transfer method^18^. Since then, many different techniques have been demonstrated for heterojunction fabrication including mechanical exfoliation and transfer^7,14,19–24^, vertical and lateral heterojunction via direct CVD growth^25–32^, and vertical heterojunction in TMD thin films^33–35^. The stacking of 2D layered materials by mechanical exfoliation is a widely used method to create van der Waals heterostructures for laboratory research. In this process the mechanically exfoliated thin layer of one TMD is transferred to another type of TMD by using intermediate polymer to form a heterojunction^14,19,36,37^. This process requires days in preparing one heterojunction device due to the complexity in placement of one material on top of the other, thus cannot be scalable, a critical requirement for semiconductor device manufacturing technologies. Selective doping via oxygen and fluorine based plasma, and radiation have also been used in mechanically exfoliated samples to fabricate lateral heterojunctions^38–40^ demonstrating proof of concepts and they are not scalable due to difficulties in achieving good yields of samples from mechanical exfoliation.

Individual layer of co-evaporation based chemical vapor deposition (CVD) grown TMDs along with post growth transfer process have been used for the fabrication of heterojunctions^41^. A few successful examples of vertically stacked TMD heterostructures have been demonstrated, however, these heterojunctions are limited to very small areas lacking a control of their location, size, and coverage hindering their scalability. The direct growth of 2D heterostructure has also been made via chemical vapor deposition (CVD) method^25–32^. The co-evaporation of metal/metal oxides and chalcogen produces many TMDs heterostructures at random locations on the growth substrate^25,28,31,42,43^. However, electrical connections to these heterojunction requires complex fabrication steps. Therefore, the scalability of the co-evaporation based CVD technique for heterojunction device fabrication is uncertain.

While large area TMD thin films, prepared via chalcogenization of metal (molybdenum, tungsten) or metal oxide films^44,45^, could be useful in the fabrication of heterojunctions, development of reliable techniques for scalable fabrication is still at its infancy. One useful technique could be the selective chemical doping of 2D TMD thin films for the fabrication of lateral heterojunctions. However, it is not known if the chemical doping can be successfully implemented in TMD thin films. In particular, benzyl viologen (BV) molecules are suggested to be promising candidates for the chemical doping of carbon nanotubes, graphene and other 2D materials^46,47^. BV is a good choice for charge transfer doping, as it has one of the lowest reduction potential among the electron-donating organic molecules^48^ and can transfer electrons to a suitable host acceptor material through a direct redox reaction, resulting in a change of carrier concentration in the host material. Therefore, doping of 2D TMD thin films by using BV molecules could change their charge carriers and transport properties and selective doping could open up the possibility of creating a band offset with undoped TMD thin films to fabricate many TMD based heterojunctions in a massively parallel fashion.

In this paper, we demonstrate an effective chemical doping of 2D TMD thin films via BV molecules and use a selective doping technique to fabricate many lateral heterojunctions simultaneously on a single chip. The large area MoS_2_ thin films were grown via low pressure sulfurization of Mo films. The electrical characterization performed before and after BV doping show that the resistance of the samples decreased by up to two orders of magnitude upon doping. X-ray photoelectron spectroscopy (XPS) measurements showed a relative shift of peak positions towards higher binding energy values for the BV doped samples suggesting n-doping of the films. The amount of doping can be controlled by sample immersion time to the BV solution. We then implemented a selective area doping method to create doped and undoped-MoS_2_ regions to fabricate many lateral heterojunctions simultaneously on a 1 cm^2^ chip. The fabricated junctions showed excellent current rectification behavior demonstrating the effectiveness and scalability of this doping method. Similar results for BV doping of MoSe_2_ thin film along with parallel fabrication of heterojunctions between doped and undoped MoSe_2_ via selective doping were also demonstrated.

## Results and discussion

**Figure 1:**
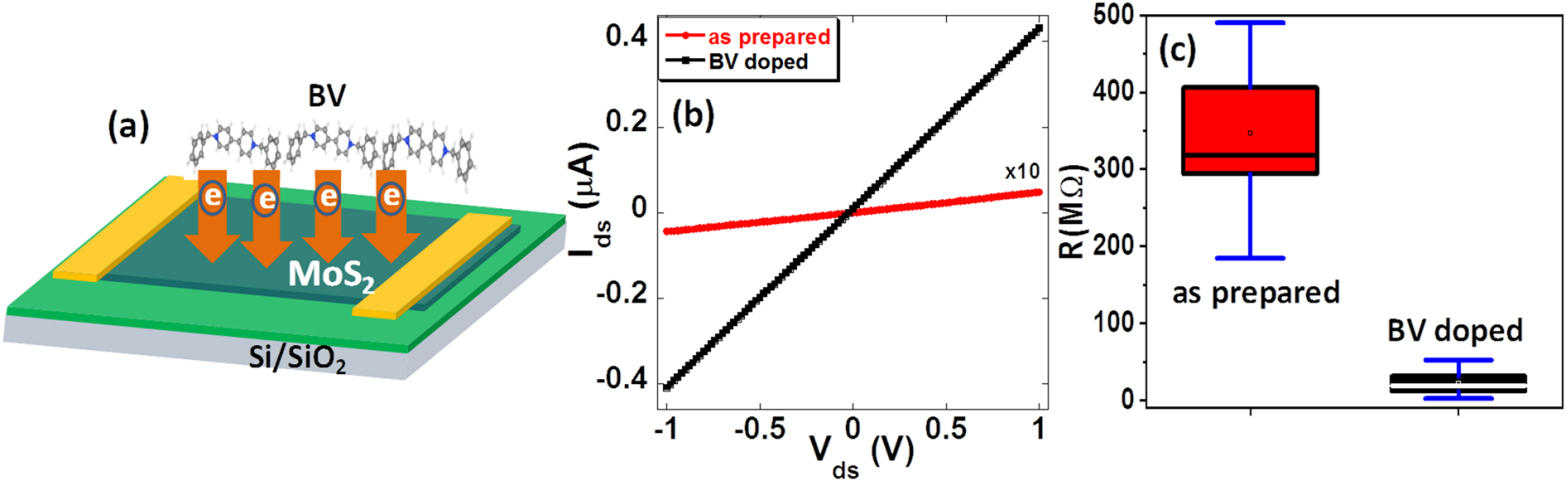
Transport properties of as prepared and BV doped MoS_2_ thin film: **(a)** schematic illustration of BV doping of a MoS_2_ device, **(b)** I–V characteristics of a representative MoS_2_ thin film device before (red) and after BV doping (black). **(c)** Box plot of resistances for 47 MoS_2_ thin film devices before and after BV doping.

Large area MoS_2_ thin films were prepared via low pressure sulfurization of molybdenum film deposited on a Si/SiO_2_ substrate, as described in the method section. Raman characterization confirmed the fabricated samples to be a few layer MoS_2_ (supplementary information figure S2). MoS_2_ devices of L = 100 µm and W = 300 µm were fabricated by defining drain source electrodes with a shadow mask. Electrical transport measurements of the undoped and BV doped devices were then carried out. The schematic diagram of a MoS_2_ device along with charge transfer process from BV molecules to MoS_2_ thin film is shown in Fig. 1a. Figure 1b shows a representative low-bias current–voltage (I_ds_ − V_ds_) characteristics of an undoped MoS_2_ sample (red curve). The resistance of this sample is calculated to be 215 MΩ. We have used highly doped Si as a back gate, however, no gate dependence was observed consistent with a few other reports^34,49–52^. We have measured 47 MoS_2_ devices fabricated on two chips. The resistance of these devices varied from 185 to 490 MΩ with an average of 347 MΩ as shown in the box plot of Fig. 1c. The measured values of resistance for our MoS_2_ devices are consistent with what has been reported in the literature^34,49–51,53,54^. After characterizing the as-prepared samples, we doped the MoS_2_ film by immersing it in BV solution for 36 h (see “Method” section for detail) and measured their electrical transport properties. Figure 1b black curve shows the I–V characteristics of the same sample represented by the red curve after BV doping. The resistance of the sample changed to 2.4 MΩ, a decrease of ~ 90 times. The significant decrease of resistance (increase of the current) after immersion of the MoS_2_ thin film into BV solution indicates that the MoS_2_ thin film is strongly doped by the BV molecules due to the transfer of electrons from BV to MoS_2_^46,55^ via surface charge transfer process resulting in an increase of charge carrier concentration. The charge transfer occurs through the redox reaction shown in supplementary Figure S1 (a) and the electron transfer mechanism can be understood by using the schematic diagram shown in supplementary Figure S1 (b). BV is known to have three oxidation states; BV^0^ (neutral molecule), BV^+^ and BV^2+^ with a reduction potential of − 0.790 V (BV^+^  →  BV^0^) and -0.332 for (BV^2+^  →  BV^+^) vs the standard hydrogen electrode (SHE). On the other hand, MoS_2_ is known to have a conduction band edge at around 0 V vs SHE^55^. The lower reduction potential of BV compared to the MoS_2_ conduction band edge gives rise to electron transfer from BV to the MoS_2_ surface which increases the electronic carrier concentration (decrease of resistance) of MoS_2_ channel.

We have measured a total of 47 MoS_2_ devices after immersing them in BV solution and found that the resistance for all the samples decreased. This is more clearly shown in the box plot of Fig. 1c. From the box plot, we see that the average resistance of as prepared MoS_2_ devices is 347 MΩ, which drops to 22 MΩ after doping. The average resistance of BV doped MoS_2_ devices is 94% lower than as prepared MoS_2_ devices. Since thin film of MoS_2_ is typically more resistive than their exfoliated and co-evaporation based CVD counterparts, the decrease in resistance by BV doping represents a significant step in the utilization of these samples in electronic devices.

**Figure 2:**
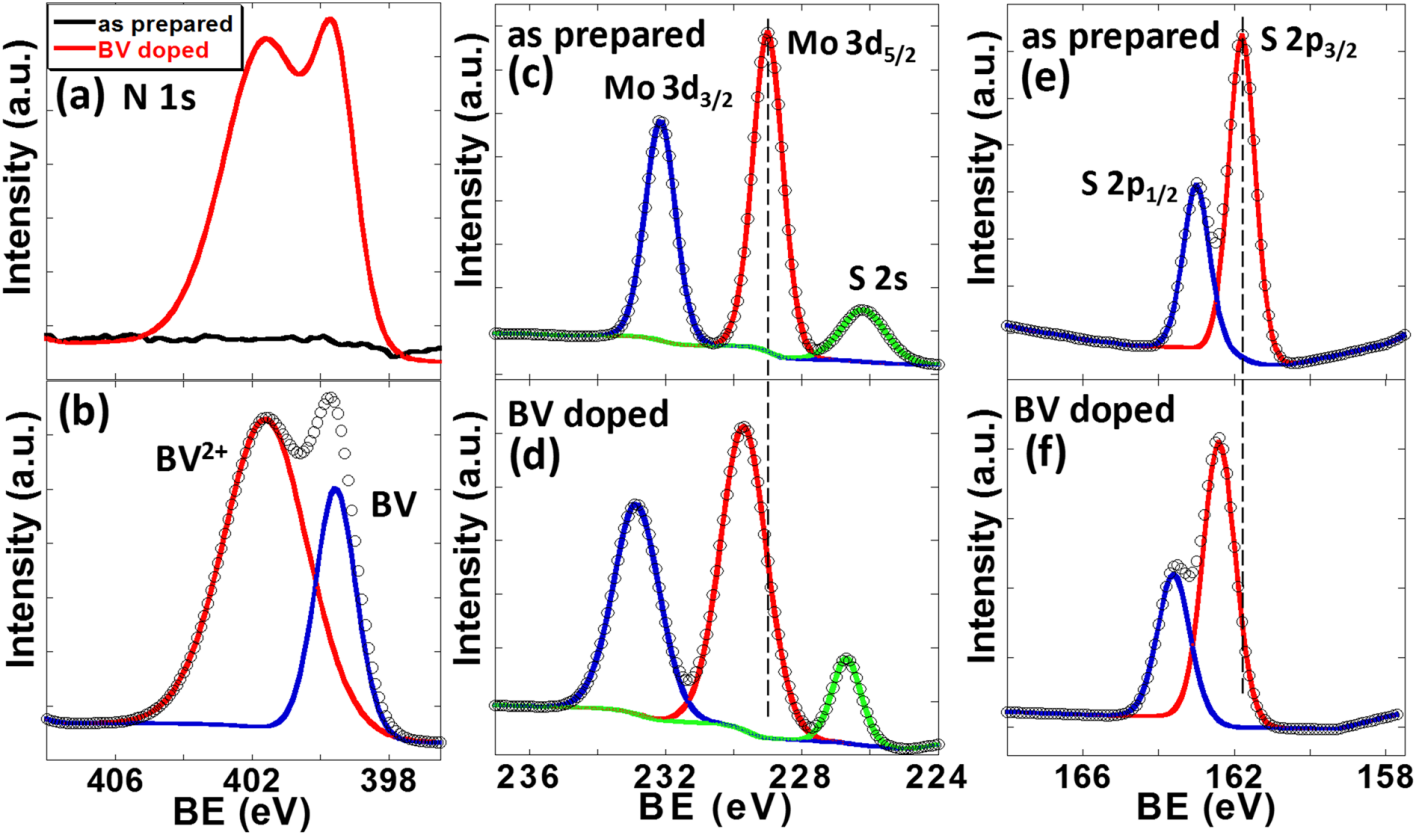
XPS measurements of MoS_2_ thin film before and after BV doping: **(a)** N 1s narrow scan of MoS_2_ film before and after BV treatment. **(b)** The deconvoluted XPS spectra of N 1s peaks of doped sample (red curve of **(a)**). Mo 3d core level XPS spectra of the **(c)** as prepared and **(d)** BV doped MoS_2_ films. The dashed line is a guide to the eye. S 2p XPS spectra of the **(e)** as prepared and **(f)** BV doped MoS_2_ films. The spectra were deconvoluted using Gaussian–Lorentzian curves. The symbols are the experimental points and the solid lines are the deconvolution of the data.

To understand the effect of BV doping on MoS_2_ thin film, X-ray photoelectron spectroscopy (XPS) measurements were performed for the as prepared and BV doped MoS_2_ samples (Fig. 2). BV doped MoS_2_ film showed a characteristic N 1s peak that was not seen in untreated MoS_2_ film as shown in Fig. 2a. The deconvoluted N 1s narrow scan of BV treated MoS_2_ shows peaks at 399.6 eV and 401.9 eV (Fig. 2b). These peaks can be assigned to BV molecule and positively charged BV molecule (BV^2+^)^56,57^. The presence of BV^2+^ peak suggest charge transfer from BV to MoS_2_ occurred (also see Figure S1) as the charge transfer will change neutral BV molecule to BV^2+^. The Mo 3d XPS spectra of as prepared MoS_2_ sample showed three prominent peaks at 226.1, 229.0 and 232.1 eV corresponding to binding energies (BEs) of S 2s, Mo 3d_5/2_ and Mo 3d_3/2_ electrons of MoS_2_, respectively (Fig. 2c) which was shifted to 226.8, 229.7 and 232.8 eV, respectively upon BV doping (Fig. 2d). The peaks were shifted towards higher binding energy values by 0.7 eV. S 2p core level XPS spectra of the same sample showed two prominent peaks with peaks position at 161.8 and 163.0 eV corresponding to S 2p_3/2_ and S 2p_1/2_ spin orbit split components of MoS_2_ respectively (Fig. 2e)^52^ which was up shifted by 0.65 eV to 162.45 and 163.65 eV, respectively (Fig. 2f). This upshift of BEs of doped MoS_2_ film indicates a relative shift of the Fermi level towards the conduction band edge suggesting n-doping of MoS_2_ films due to charge transfer by BV molecules, consistent with our electrical transport measurements^58,59^. Raman spectroscopy was also used to characterize BV doped MoS_2_ samples (supplementary information Figure S2), however, BV doping only showed a minimal effect on the vibrational modes of the samples.

**Figure 3:**
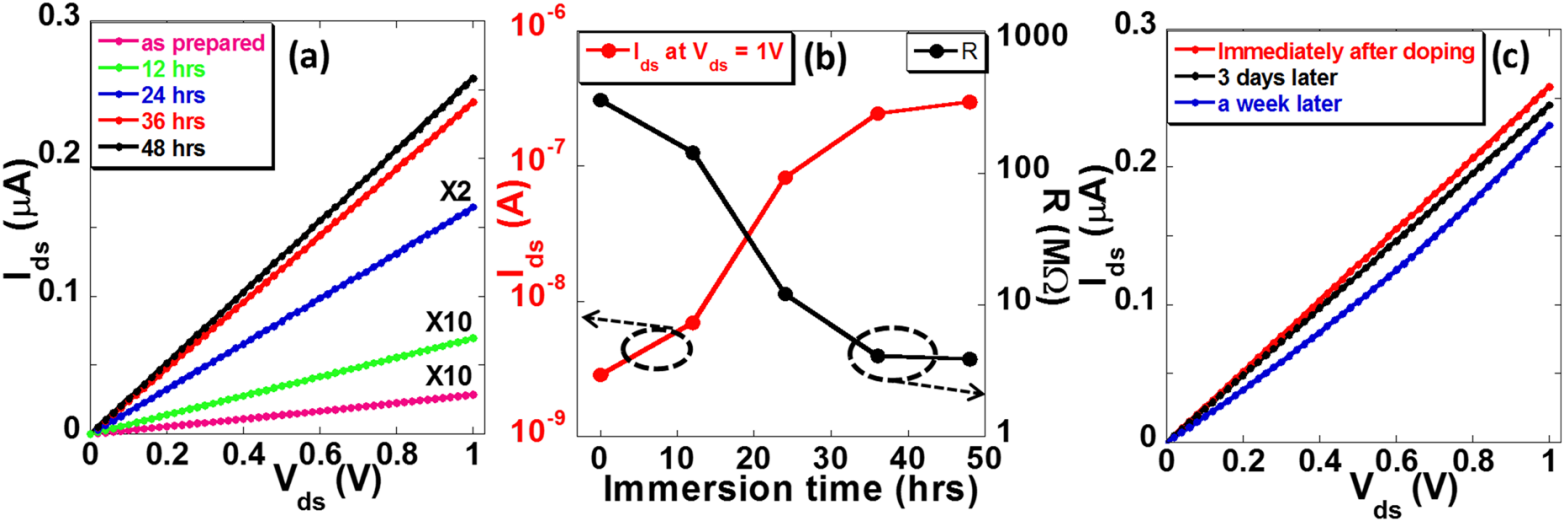
Doping by varying the immersion time and stability of the BV doped MoS_2_ thin film: **(a)** I–V characteristics of a BV doped MoS_2_ device with different immersion time. **(b)** Current and resistance variation of the MoS_2_ device with varying immersion time in BV solution. The current was recorded at V_ds_ = 1 V and the resistance was calculated from linear fit of the I–V curve. **(c)** I–V characteristics to show the stability of the doped MoS_2_ device. The I–V characteristics were measured immediately after doping, after 3 days and after a week.

We also tested whether the amount of doping can be controlled by varying the immersion time of MoS_2_ in the BV solution and measuring the electrical properties of the MoS_2_ device after each immersion time. Figure 3a shows I–V characteristics of a representative BV doped MoS_2_ device with immersion time varying from 12 to 48 h. The drain current (resistance) increases (decreases) with increasing immersion time and then saturates. We observed the current at V_ds_ = 1 V increased from ~ 2.9 × 10^–9^ A to 7.0 × 10^–9^ A after 12 h immersion of the MoS_2_ device in BV solution resulting in a decrease of resistance from 360 MΩ to 142 MΩ. With further 12 h (total 24 h) soaking of the MoS_2_ device in BV solution, the current increased to ~ 8.2 × 10^–8^ A (resistance decreased to 12.2 MΩ). The current further increased to ~ 2.5 × 10^–7^ A with an additional 12 h (total 36 h) of immersion resulting in a decrease of resistance to ~ 4.1 MΩ. With further immersion of the MoS_2_ device for another 12 h (total of 48 h) in BV solution, resistance decreased to 3.9 MΩ which is a small fraction of change compared to the MoS_2_ device with 36 h immersion. The variation of current and resistance with immersion time is more clearly shown in Fig. 3b. It can be seen that the current increases rapidly for the first 24 h and then increases slowly to 36 h. After 36 h, the current almost saturates. When MoS_2_ device is immersed in BV solution, BV molecules adsorb on the MoS_2_ surface. The BV donates an electron to MoS_2_ and changes to another oxidation state (BV^+^). The BV^+^ further donates an electron to MoS_2_ surface and becomes BV^2+^. More BV molecules adsorb on the MoS_2_ surface with increase of immersion time which causes more charges transfer from BV to MoS_2_. When the MoS_2_ surface is saturated by BV^2+^ molecules, further charge transfer stops. Notably, the BV doped MoS_2_ thin film devices are relatively stable with only minimal change in the current over a period of a week (Fig. 3c) The resistance changed from 3.9 MΩ to 4.6 MΩ after a week of ambient exposure.

**Figure 4:**
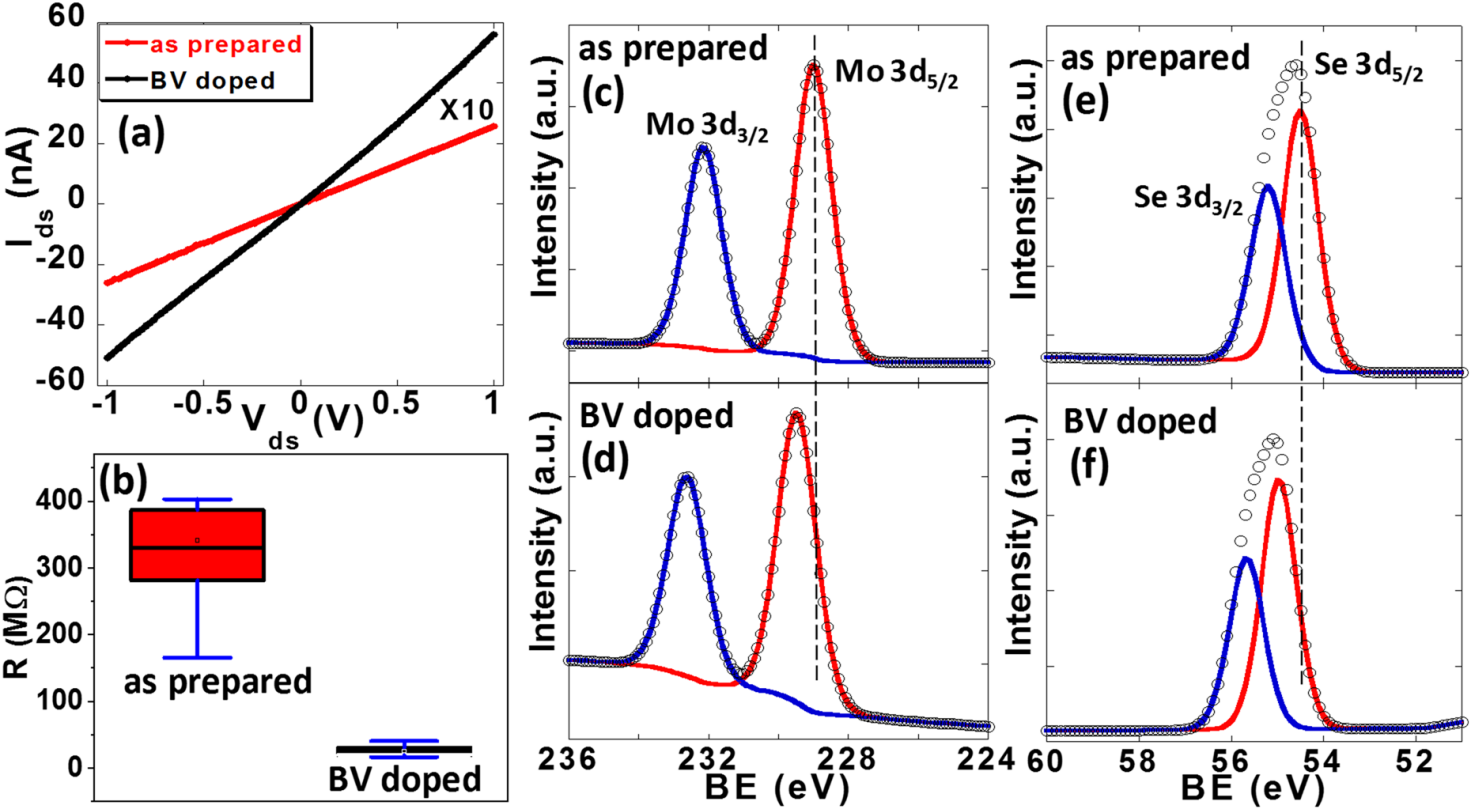
Transport properties and XPS spectra of as prepared and BV doped MoSe_2_ thin films: **(a)** I–V characteristics of a as-prepared MoSe_2_ thin film device before (red curve) and after BV doping (black curve). **(b)** Box plot of resistance of 27 MoSe_2_ devices before and after BV doping. Mo 3d core level XPS spectra of **(c)** a as-prepared and **(d)** BV doped MoSe_2_ films. The dashed line is a guide to the eye. Se 3d XPS spectra of **(e)** as prepared and **(f)** BV doped MoSe_2_ films. The spectra were deconvoluted using Gaussian–Lorentzian curves. The symbols are the experimental points and the solid lines are the deconvolution of the data.

To test effectiveness of BV doping on other TMD materials, we studied the influence of BV molecules on the transport properties of MoSe_2_ thin film. The MoSe_2_ film was prepared via low pressure selenization of Mo film. Raman characterization was carried out to confirm that the synthesized film was a few layer MoSe_2_ (supplementary information Figure S3). Figure 4a shows I–V curve of a representative undoped MoSe_2_ device (red curve). The resistance of this sample is calculated to be 388 MΩ. We have measured 27 MoSe_2_ devices that were fabricated on the same chip and the resistance of the devices varies from 150 MΩ to 410 MΩ with an average of 320 MΩ as shown in the box plot of Fig. 4b. The MoSe_2_ samples were then immersed in BV solution for 36 h for charge transfer doping process following which the electrical characterizations were carried out. Figure 4a black curve shows the I-V characteristics of the same sample represented by the red curve after BV doping. The resistance of this sample changed to 19 MΩ. We have measured all 27 samples after doping and found that the resistance of all the samples varies from 17 to 55 MΩ with an average of 33 MΩ. This is shown in Fig. 4b. The average resistance decreased by ~ 90% after BV doping of MoSe_2_ devices. This suggests that, similar to MoS_2_ thin film, BV molecules can effectively dope MoSe_2_ thin film by transferring electrons from BV molecules to MoSe_2_. XPS measurement of MoSe_2_ films further confirms the charge transfer based doping. For as prepared MoSe_2_ thin film, the XPS peaks correspond to Mo 3d_5/2_ and 3d_3/2_ were observed at binding energies 229.1 and 232.2 eV respectively (Fig. 4c). In addition, the peaks observed at the binding energies of 54.5 and 55.3 eV in the Se 3d spectra can be assigned to the Se 3d_5/2_ and Se 3d_3/2_ orbitals of MoSe_2_ (Fig. 4e)^60^. Upon doping the MoSe_2_ thin film, both Mo 3d and Se 3d peaks were shifted by 0.5 eV to the higher binding energy values (Fig. 4d, f). This upshift of BEs indicates a relative shift of the Fermi level toward the conduction band edge suggesting n-doping of the films^58,59^. This is consistent with MoS_2_ XPS measurements.

**Figure 5:**
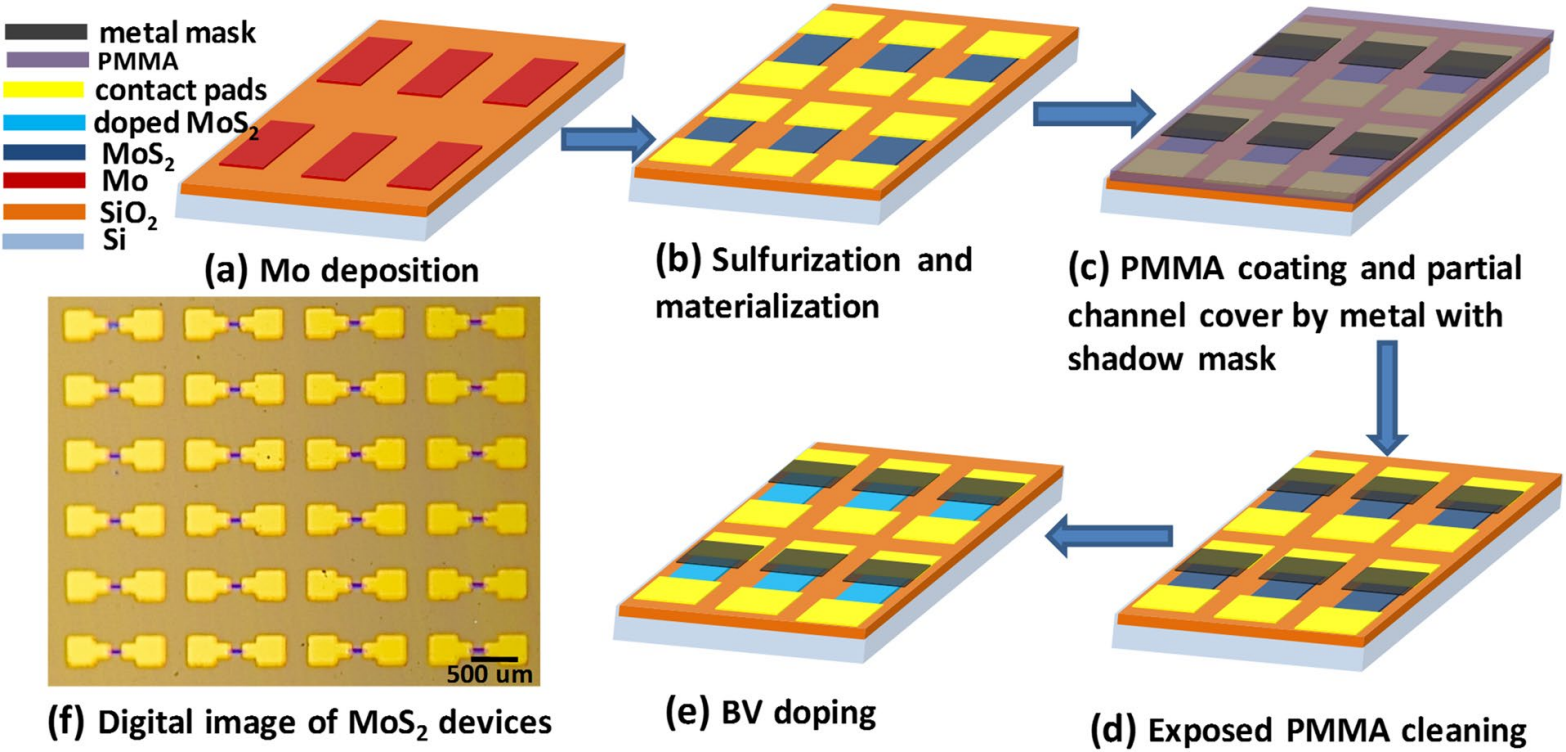
**(a)**–**(e)** Schematic flow chart showing the different fabrication steps of MoS_2_ thin film heterojunctions by selective BV doping technique. Lateral heterojunctions were formed at the junctions of undoped MoS_2_ and doped MoS_2_ regions. **(f)** Digital image (partial) of MoS_2_ heterojunction devices.

A selective doping process was developed to fabricate many lateral heterostructure devices simultaneously on a 1 cm^2^ chip. Figure 5 shows the schematic diagram of the fabrication steps. First, patterned molybdenum (Mo) film was deposited using a shadow mask (Fig. 5a) followed by the low pressure sulfurization to grow MoS_2_ thin film. Cr/Au metal contacts were deposited (Fig. 5b) using another shadow mask. For the selective doping, we coated poly-methyl methacrylate (PMMA) on the MoS_2_ thin film and deposited aluminum metal through another shadow mask to cover about half of the MoS_2_ channel (Fig. 5c). The regions of the PMMA left uncovered with aluminum was etched away using oxygen plasma to partially expose the channel of MoS_2_ thin film devices for BV doping (Fig. 5d). The entire substrate was then immersed into BV solution for 36 h. The regions covered by aluminum/PMMA are protected and left unexposed to BV molecules. Only the exposed regions of the MoS_2_ channel were doped by BV solution due to charge transfer process. This process creates doped and undoped sides of the MoS_2_ channel (Fig. 5e). As a result, heterojunctions are created at the junctions of doped and undoped MoS_2_ regions. Since many samples can be simultaneously patterned in the same batch, this process is promising for creating scalable lateral heterojunctions. A digital image showing many heterojunctions created in the same chip is presented in Fig. 5f. A high magnification optical image of a single MoS_2_ heterojunction device is shown in supplementary information Figure S4. The heterojunction properties were confirmed by the electrical transport measurements.

**Figure 6:**
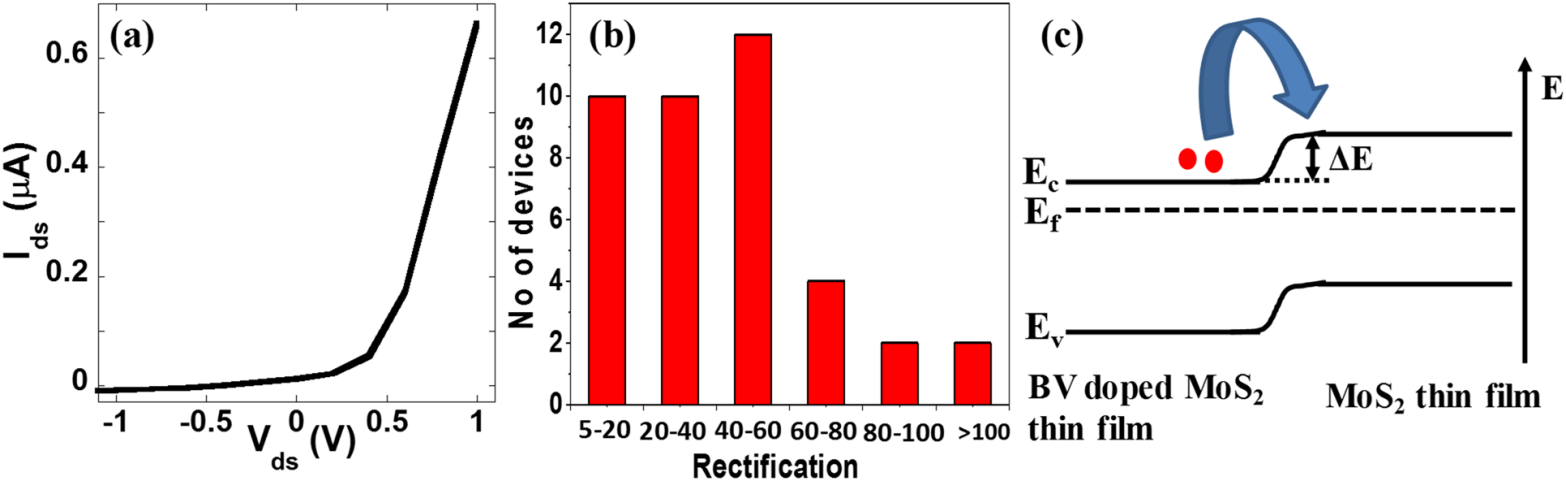
Current rectification characteristics of MoS_2_ heterojunction after selective chemical doping: **(a)** I–V characteristics of a MoS_2_ heterojunction device prepared by selective BV doping. **(b)** Statistics of rectification behavior of 40 MoS_2_ heterojunction devices simultaneously fabricated in a single chip. **(c)** Schematic band diagram showing band offset (Δ*E*) between the undoped and doped MoS_2_ regions.

Figure 6a shows I–V characteristics of a representative selectively doped MoS_2_ lateral heterojunction device measured at room temperature. The I–V curve shows nonlinear diode like characteristics, with a very high current for forward bias and negligible current for reverse bias, expected for a heterojunction device. This device did not show reverse bias breakdown for up to − 1.0 V. The rectification ratio (I_forward_/I_reverse_) of this device is calculated to be ~ 78. As the individual MoS_2_ devices did not show any gate dependence, the heterojunction devices did not show any gate dependence characteristics either when highly doped Si was used as back gate. In order to ensure that the measured electrical properties are originated from the fabricated heterojunctions, we monitored the effect of the fabrication process by measuring I–V curves after every fabrication step (supplementary information figure S5). Current rectification behavior was not observed before immersing the samples into the BV solution. In addition, the I–V curves of as fabricated and doped devices are linear (Fig. 1b), suggesting that the observed current rectification behavior is not coming from Schottky contacts between MoS_2_/metal electrodes. This observation confirms the formation of the lateral heterojunctions of MoS_2_ by selective doping method. We have fabricated a total of 48 MoS_2_ lateral junction devices simultaneously on the same 1 cm^2^ chip and measured their electrical transport properties. Out of them, 40 devices showed current rectification behavior. Figure 6b summarizes the rectification behavior of all the devices. The lateral junction devices exhibited current rectification behavior with a rectification ratio of up to two orders of magnitude. Notably, the fabricated heterojunction devices are relatively stable and showed minimal change in I–V curve and rectification ratio over a period of a week (supplementary information figure S6) consistent with the observed air stability of BV doped MoS_2_ thin film device presented in Fig. 3c. Our result can be explained by the energy-band diagram of Fig. 6c that illustrates the band profiles and band alignment between the undoped MoS_2_ and BV doped MoS_2_ regions. The work function of the pristine MoS_2_ has been reported to be in the range of 4.6–4.9 eV^61–64^. Although, the exact work function of BV doped MoS_2_ is not known, we expect this to be lower than MoS_2_ due to the increased carrier concentration by doping as the work function is known to be modulated by p or n-doping^5,65^. This is further supported by the XPS measurements where the characteristics peaks were shifted towards higher binding energy values (Fig. 2) indicating n-doping of the thin film by BV molecules. The n-doping causes a relative shift of fermi level towards the conduction band of MoS_2_ resulting in a decrease of work function of the doped part of the channel. This would create a band offset (∆E) between the doped and undoped regions of MoS_2_ resulting in the formation of lateral heterojunctions. This band offset prevents the flow of charge carriers at the negative bias voltages and leads to a rectification behavior. Similar selective doping method has been implemented to simultaneously fabricate many MoSe_2_ heterojunction devices on a 1 cm^2^ chip which also showed excellent current rectification behavior, demonstrating the scalability of the technique (supplementary information figure S7).

## Conclusion

We demonstrated a simple technique for the doping of TMD thin films and used selective doping for scalable fabrication of lateral heterojunctions. We show for the first time that when MoS_2_ and MoSe_2_ thin films, grown via low pressure sulfurization and selenization of Mo films, are immersed into BV solution, the resistance decreases by up to two-orders of magnitude due to the surface charge transfer based doping. XPS measurements show a relative shift of characteristics peak positions towards higher BEs for the doped samples indicating an n-doping of the films by BV molecules. The amount of doping can be controlled by varying the immersion time of the thin film devices in BV solution. Using a simple patterning technique, we implemented selective doping of MoS_2_ samples and fabricated many MoS_2_ lateral heterojunction devices simultaneously on a 1 cm^2^ chip. The electrical transport measurements performed across MoS_2_ junction exhibit current rectification behavior with a rectification ration of up to two orders of magnitude due to the band offset created by selective doping. Similar heterojunction devices via selective doping was also demonstrated in MoSe_2_ thin film which exhibits current rectification behavior. The doping method presented here provides an efficient way to control the electrical properties of TMDs based thin film and when combined with the selective doping technique to fabricate scalable heterostructure devices.

## Methods

### MoS_2_ and MoSe_2_ film growth

Molybdenum (Mo) film of 6 nm thickness was deposited by an electron beam evaporator at a low evaporation rate of 0.05 Å/s on a Si/SiO_2_ substrates with 250 nm of thermal oxide layer. The substrate was then placed in the center zone of one-inch quartz tube furnace (Barnstead International F79300 Tube Furnace) and an alumina boat containing sulfur (S) powder was placed in the furnace upstream side. The system was pumped down to a base pressure of ∼30 mTorr and purged argon (Ar) gas to remove oxygen and water vapor. The Ar gas flow of ∼130 standard cubic centimeters per minute (sccm) was maintained during entire growth process. The furnace was heated to the growth temperature of 800 °C with ramping rate of 15°/min and was maintained at the growth temperature for 55 min. The sulfur vapor was carried by Ar gas to the central zone where it reacts with the Mo to form MoS_2_ films. The chamber was allowed to cool down to room temperature naturally after growth. MoSe_2_ film was prepared by selenization of the 2 nm Mo metal film using same method as MoS_2_ film preparation.

### Raman characterization

Raman characterization was performed using a Witec alpha 300 RA confocal Raman microscope with laser source of excitation wavelength of 532 nm and power of < 1 mW in ambient conditions at room temperature. A 100 × objective was used to focus the laser beam at a spot. Raman emission was collected and dispersed by a grating of 1,800 lines-per-mm with the data accumulation duration of 3 s.

### XPS characterization

XPS measurements of the TMD films were performed using a Thermo Scientific (Escalab Xi) XPS system with a monochromatic Al Kα radiation source. Pass energy of 20 eV with 0.1 eV scanning step was used for photoelectron detection. XPS spectra were taken onto the sample surface with a scan area 300 × 300 µm^2^ and carbon (C) 1 s reference line at the binding energy of 284.8 eV was used to calibrate the charging effect.

### Device fabrication and transport characterization

For the electrical transport characterization of as prepared and BV doped MoS_2_ and MoSe_2_ films, 5 nm/40 nm Cr/Au electrodes were patterned on top of MoS_2_ and MoSe_2_ films using a shadow mask. The electrodes were deposited with a deposition rate of 0.05 Å/s at a base pressure of 5 × 10^–7^ mBar. The electrical transport measurements of the devices were performed in a probe station using a current preamplifier (DL instruments 1211) interfaced with Lab View program. All electrical measurements were carried out at room temperature in ambient conditions.

### Synthesis of benzyl viologen (BV) and doping

25 mg benzyl viologen dichloride (Sigma-Aldrich) was dissolved into 5 ml of nanopure DI water. 5 ml of toluene was added to benzyl viologen dichloride and water to make a bilayer solution. ~ 3.7 gm of Sodium borohydride (Sigma-Aldrich) was added to the water/toluene bilayer solution. The solution was then kept for one day to form benzyl viologen (BV). The BV accumulated in the toluene solution. The toluene solution with BV molecules was carefully extracted using pipette. The MoS_2_ and MoSe_2_ devices was immersed into the extracted BV solution for doping of the thin films. The doping was performed by immersion of the MoS_2_ and MoSe_2_ devices into the BV solution for 12 to 48 h. The samples were then taken out of the solution and allowed to dry naturally.

## Supplementary information

Supplementary Information.

## Data Availability

The data that support the findings of this study are available from the corresponding author upon reasonable request.
