# Supplementary material for: Scalable lateral heterojunction by chemical doping of 2D TMD thin films
Source: Sci Rep. 2020 Jul 31;10:12970. doi: 10.1038/s41598-020-70127-6 (PMC7395794; doi:10.1038/s41598-020-70127-6)
Supplement: Supplementary file 1 — Supplementary Information. [file 41598_2020_70127_MOESM1_ESM.docx]

**Supplementary information for**

**Scalable Lateral Heterojunction by Chemical Doping of 2D TMD Thin Films**

Bhim Chamlagain^1^, Sajeevi S. Withanage^1^, Ammon C. Johnston^1^, and Saiful I. Khondaker^1,2,*^

^1^ NanoScience Technology Center and Department of Physics, University of Central Florida, Orlando, FL 32826, USA.

^2^ School of Electrical Engineering and Computer Science, University of Central Florida, FL 32826, USA.

*saiful@ucf.edu

**S1.** **Doping mechanism of MoS_2_ thin film by benzyl viologen molecule**


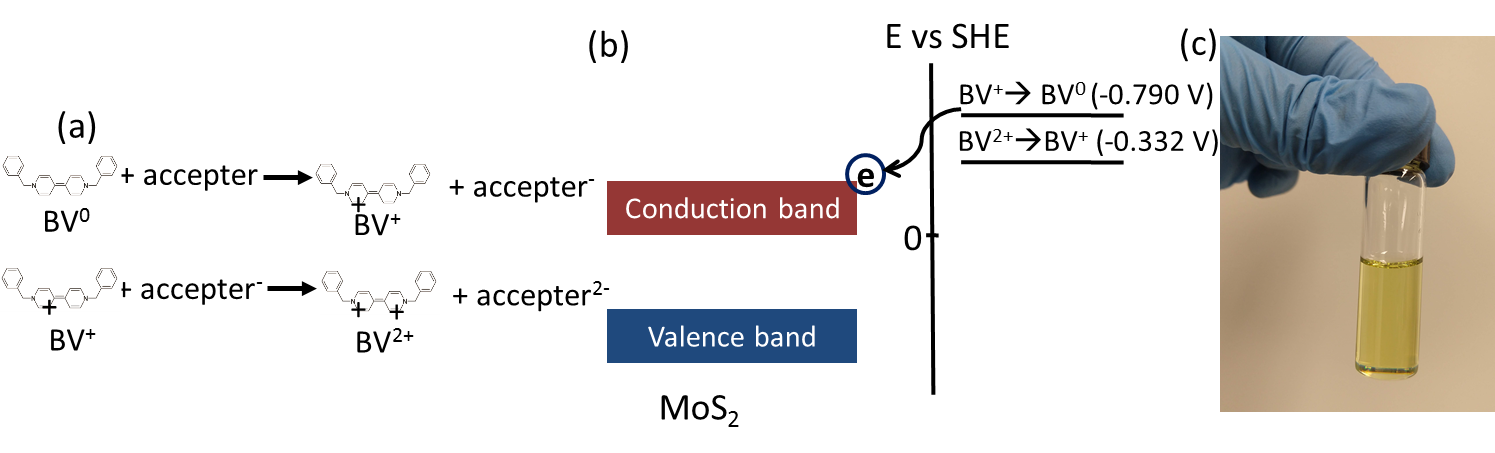


**Figure S1. Doping mechanism of MoS_2_ thin film by benzyl viologen (BV) molecule:** (a) Redox reactions of a BV molecule when it comes in contact with an acceptor material (b) Schematic illustration of energy band diagram of MoS_2_ and BV molecule. MoS_2_ conduction band edge is ~ 0V of potential vs SHE (standard hydrogen electrode). Reduction potential of BV is lower than the conduction band edge of MoS_2_. This allows for spontaneous transfer of an electron from BV to MoS_2_ when the BV molecule adsorbs on the MoS_2_ surface. (c) Extracted benzyl viologen (BV) solution from biphasic solution of water/toluene. The yellow BV solution was used for doping of both MoS_2_ and MoSe_2_ films and fabrication of heterojunctions.

**S2.** **Raman characterization of MoS_2_ thin film before and after BV doping**


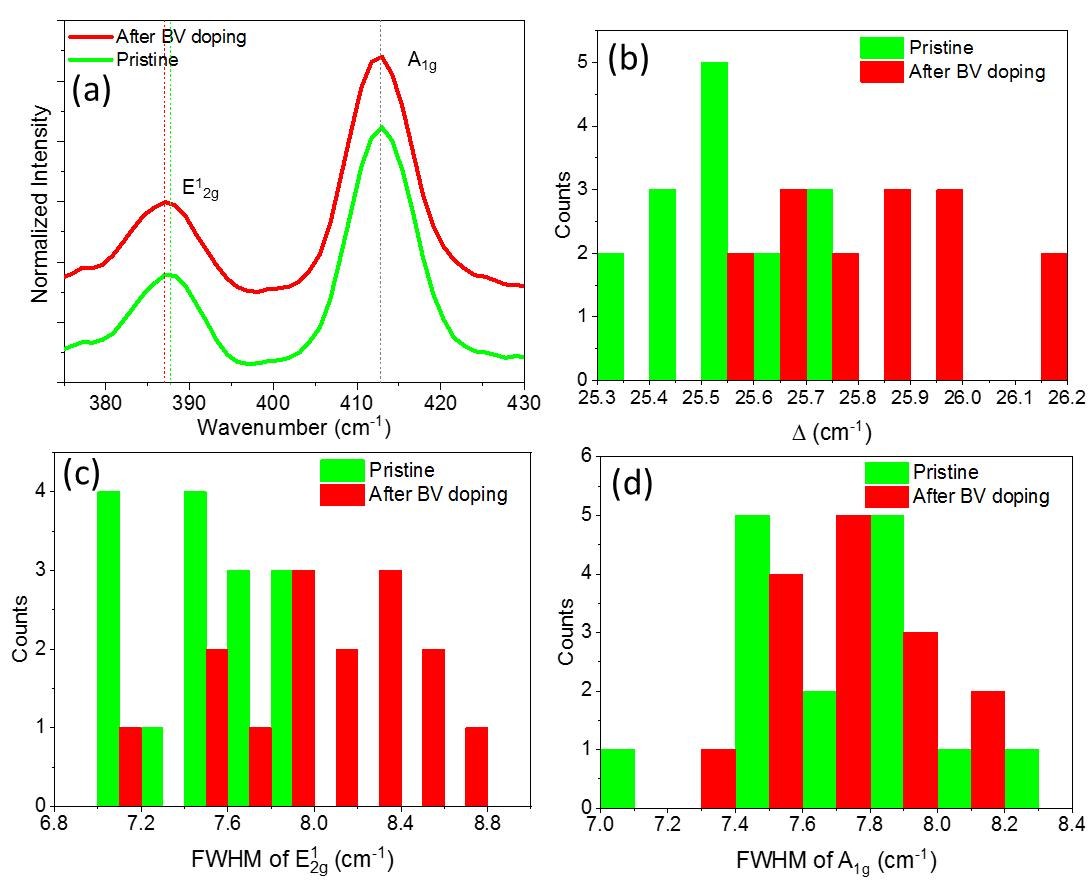


**Figure S2.** **Raman characterization of MoS_2_ thin films before and after BV doping:** (a) Raman spectra of as prepared and BV doped MoS_2_ films. (b) Statistics of Δ (difference of E^1^_2g_ and A_1g_ peaks) of MoS_2_ samples before and after doping. (c) Statistics of full width half maximum (FWHM) of E^1^_2g_ peaks for as prepared and BV doped MoS_2_ samples. (d) Statistics of full width half maximum (FWHM) of A_1g_ peaks of for as prepared and BV doped of MoS_2_ samples.

MoS_2_ samples were characterized by Raman spectroscopy before and after doping. The Raman spectra were taken by focusing the laser in between drain source electrodes of 15 MoS_2_ devices. The spectra were recorded by averaging over 2 µm^2^ scan area. Figure S2 (a) shows the Raman spectra of a representative MoS_2_ sample before and after doping. We observed the characteristics E^1^_2g_ and A_1g_ peaks of as-prepared MoS_2_ film at 387.2 cm^-1^ and 412.7 cm^-1^ with a position difference of 25.5 cm^-1^ while the same peaks were observed at 386.9 cm^-1^ and 412.7 cm^-1^ after BV doping with a position difference of 25.8 cm^-1^. Figure S2 (b) shows the statistical variation of Δ of 15 MoS_2_ devices before and after doping. The variation of the Δ lies in between 25.3 cm^-1^ to 25.8 cm^-1^ with an average of 25.5 cm^-1^. The values of Δ of the as prepared MoS_2_ films are consistent with the large area MoS_2_ film prepared by similar method.^1-6^ On the other hand, the statistical variation of Δ of BV doped MoS_2_ samples lies in between 25.6 cm^-1^ to 26.2 cm^-1^ with an average value of 25.8 cm^-1^.

Figure S2 (c) and S2 (d) show the analyzed FWHM of the E^1^_2g_ and A_1g_ peaks of the MoS_2_ samples respectively before and after doping. We observed the values of FWHM of E^1^_2g_ peak of as prepared MoS_2_ samples lie in between 7 to 8 cm^-1^ and A_1g_ peak lie in between 7 to 8.3 cm^-1^ while the corresponding average values were 7.5 cm^-1^ and 7.7 cm^-1^ respectively. After BV doping, the values of FWHM of E^1^_2g_ peak lie in between 7.1 to 8.8 cm^-1^ with an average of 8.0 cm^-1^ while A_1g_ peak lie in between 7.3-8.2 cm^-1^ with an average of 7.7 cm^-1^. It has been reported that for mechanically exfoliated MoS_2_ flakes, the FWHM of A_1g_ peak increased from 5.2 to 8 cm^‑1^ with a small shift (~1 cm^-1^) upon doping by BV molecules which was attributed to the phonon mode softening.^7,8^ In our MoS2 thin film samples, we have not observed any significant broadening of peaks or any shift in A_1g_ peaks. We do not know the exact reason for not observing Raman shift or broadening of A_1g_ peaks in our samples, however, we speculate that this may have to do with the orientation and intrinsic disorder of the MoS_2_ thin film in comparison to their exfoliated and CVD grown counterparts.

**S3.** **Raman characterization of MoSe_2_ thin films before and after BV doping**


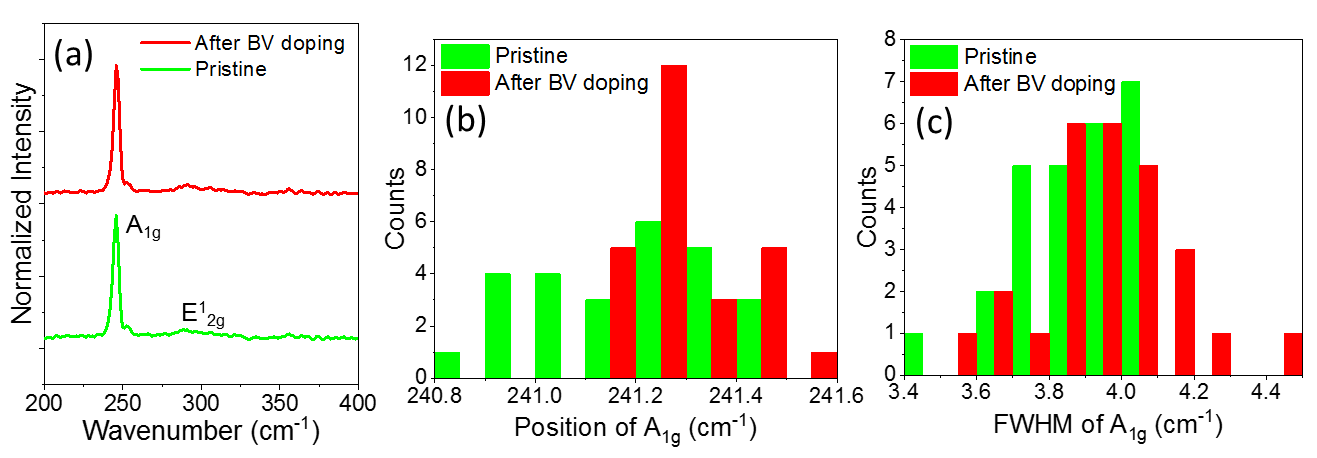


**Figure S3.** **Raman characterization of MoSe_2_ thin film before and after BV doping:** (a) Raman spectra of as prepared and BV doped of MoSe_2_ film. (b) Statistics of A_1g_ peak position of MoSe_2_ samples before and after doping. (c) Statistics of full width half maximum (FWHM) of A_1g_ peak of the Raman spectra for as prepared and BV doped MoSe_2_ samples.

Raman spectroscopy was used to characterize the as prepared and BV doped MoSe_2_ film. The results are presented in figure S3. The spectra were obtained by focusing the laser at a location of channel in between drain source electrodes of MoSe_2_ devices and averaging over 2 µm^2^ scan area. We observed the characteristics Raman spectrum of MoSe_2_ with prominent A_1g_ peak.^9^ We found that average value of A_1g_ peak for as prepared MoSe_2_ samples was at 241.2 cm^-1^ which showed a small shift to 241.3 cm^-1^ after doping. We also observed that the average FWHM of A_1g_ peak was 3.9 cm^-1^ for as prepare samples which changed to 4.0 cm^-1^ after doping.

**S4.** **Optical image of MoS_2_ heterojunction devices**


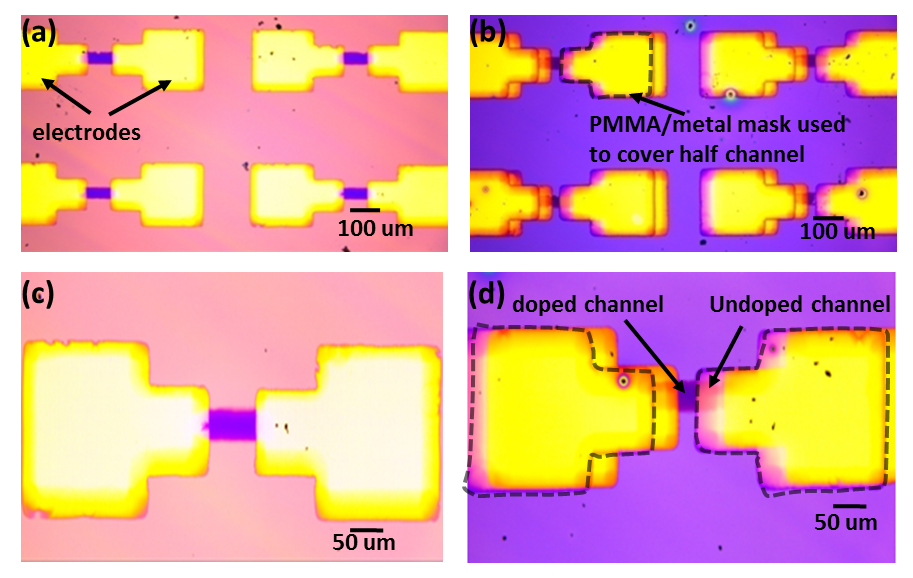


**Figure S4. Optical image of MoS_2_ heterojunction device:** Optical images of (a) MoS_2_ devices before doping and (b) PMMA/metal mask that was used to cover part of the channel which was not doped. The uncovered part was used for selective doping by BV molecules for heterojunction formation. (c) and (d) show optical images of a single MoS_2_ and heterojunction device, respectively. The black doted lines in figures S4(b) and (d) indicates the PMMA/metal mask position. Both doped and undoped channel is marked in figure S4(d). About half of the MoS_2_ channel was left open for BV doping while the other half of the channel which was covered by the PMMA/metal mask which was undoped to create a heterojunction at the junction of doped and undoped MoS_2_ regions.

**S5.** **I-V characteristics of MoS_2_ devices at different steps of heterojunction fabrication process**


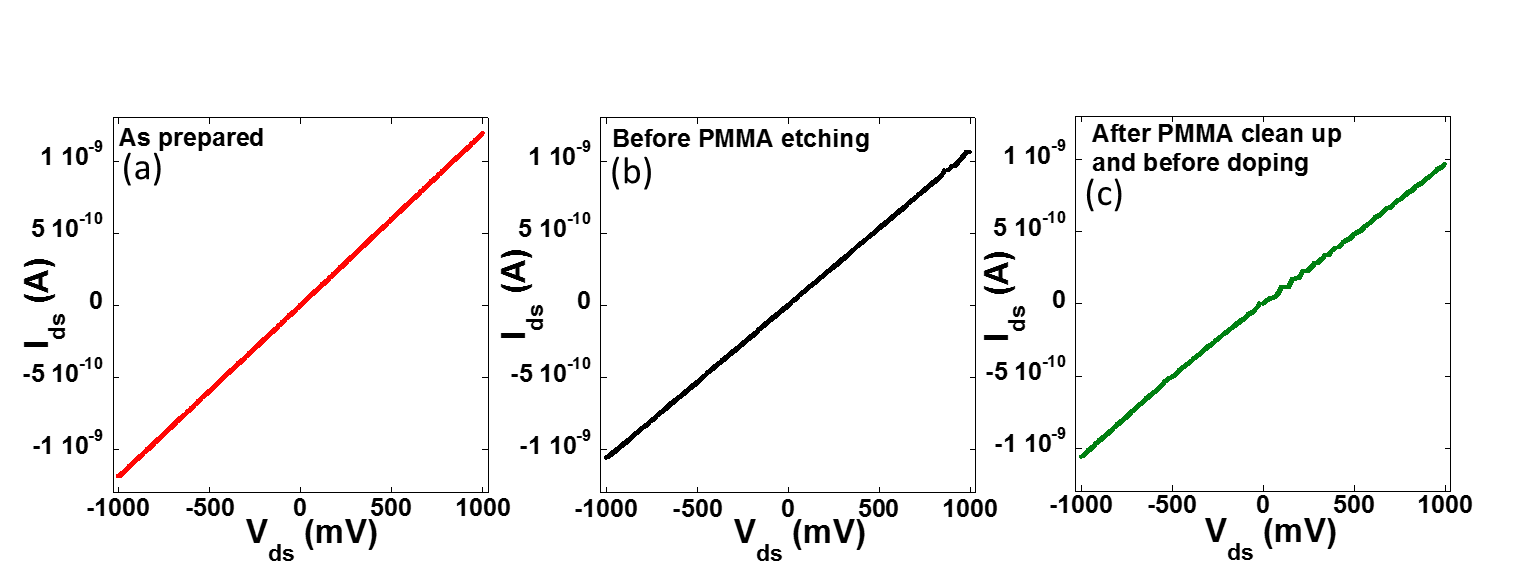


**Figure S5.** **I-V characteristics of a MoS_2_ device at different steps of heterojunction fabrication process** (a) I-V characteristics of a as-prepared MoS_2_ device. (b) I-V characteristics of the MoS_2_ device after spinning PMMA and selective patterning by shadow mask. (c) I-V characteristics of the MoS_2_ device after cleaning exposed PMMA before doping.

I-V characteristics of the heterojunction devices were measured after each fabrication step to monitor the effects of chemicals and plasma used. Figure S5 (a) shows the I-V characteristics of a representative as-prepared MoS_2_ device. The device was coated with PMMA to cover about half of the channel by aluminum with shadow mask patterning. The I-V characteristics was measured after PMMA coating. Figure S5 (b) presents the I-V characteristics of the MoS_2_ device after shadow mask patterning. The exposed part of the PMMA was cleaned by using oxygen plasma with precisely monitoring etching rate. After PMMA cleaning, I-V characteristics of the MoS_2_ device was measured in the same configuration. Figure S5 (c) depicts the I-V characteristics of the MoS_2_ device after cleaning the exposed part PMMA. The measured I-V characteristics remains unchanged after each fabrication steps without any appreciable change of the resistance.

**S6. Air stability of MoS_2_ heterojunction**

**Figure S6. Air stability of MoS_2_ heterojunction.** I-V characteristics to show the stability of MoS_2_ heterojunction device.

To test stability of the heterojunction, I-V characteristics of the heterojunction device was measured after air exposure. Figure S6 presents I-V characteristics of a representative MoS_2_ heterojunction device measured immediately after the BV doping, after 3 days and after a week of ambient exposure. The MoS_2_ heterojunction device is relatively stable and have same rectification ratio over a period of a week.

**S7. Rectification behavior of a MoSe_2_ heterojunction**

**Figure S7. Rectification characteristics of a MoSe_2_ heterojunction after selective chemical doping.** I-V characteristics of a MoSe_2_ heterojunction device prepared by selective BV doping technique.

MoSe_2_ heterojunctions were fabricated using the same technique used for MoS_2_ that was described in figure 5. After selective patterning of the MoSe_2_ channel, the substrate was immersed in BV solution to create doped and undoped MoSe_2_ regions. The band offset created between the doped and undoped regions of the devices forms a lateral heterojunction. To conform the formation of junctions due to selective doping, I-V characteristics were measured across the junctions. Figure S7 shows the I-V characteristics of a representative MoSe_2_ heterojunction device after selective doping method by simple patterning the MoSe_2_ channel. The I-V curve shows nonlinear characteristics with high current for forward bias and negligible current for reverse bias. The rectification ratio (I_forward_/I_reverse_) of the device is calculated to be ~ 41. The observed current rectification shows the formation of lateral heterojunction at the junction of doped and undoped regions of the MoSe_2_ device.

**References**

1. He, T. et al. Synthesis of large-area uniform MoS_2_ films by substrate-moving atmospheric pressure chemical vapor deposition: from monolayer to multilayer. *2D Mater.* **6**, 025030, doi:10.1088/2053-1583/ab0760 (2019).

2. Robertson, J., Liu, X., Yue, C., Escarra, M. & Wei, J. Wafer-scale synthesis of monolayer and few-layer MoS_2_ via thermal vapor sulfurization. *2D Mater.* **4**, 045007, doi:10.1088/2053-1583/aa8678 (2017).

3. Gatensby, R. et al. Controlled synthesis of transition metal dichalcogenide thin films for electronic applications. *Appl. Surf. Sci.* **297**, 139-146, doi:10.1016/j.apsusc.2014.01.103 (2014).

4. Siegel, G., Venkata Subbaiah, Y. P., Prestgard, M. C. & Tiwari, A. Growth of centimeter-scale atomically thin MoS_2_ films by pulsed laser deposition. *APL Mater.* **3**, 056103, doi:10.1063/1.4921580 (2015).

5. Yang, H. et al. Highly Scalable Synthesis of MoS_2_ Thin Films with Precise Thickness Control via Polymer-Assisted Deposition. *Chem. Mater.* **29**, 5772-5776, doi:10.1021/acs.chemmater.7b01605 (2017).

6. Agafonov, V. et al. Single Variable Defined Technology Control of the Optical Properties in MoS_2_ Films with Controlled Number of 2D-layers. *Nanotechnology* **31**, doi:10.1088/1361-6528/ab4753 (2019).

7. Kiriya, D., Tosun, M., Zhao, P., Kang, J. S. & Javey, A. Air-stable surface charge transfer doping of MoS_2_ by benzyl viologen. *J. Am. Chem. Soc.* **136**, 7853-7856, doi:10.1021/ja5033327 (2014).

8. Wang, W. et al. Surface charge transfer doping of monolayer molybdenum disulfide by black phosphorus quantum dots. *Nanotechnology* **27**, 505204, doi:10.1088/0957-4484/27/50/505204 (2016).

9. Kong, D. et al. Synthesis of MoS_2_ and MoSe_2_ films with vertically aligned layers. *Nano Lett.* **13**, 1341-1347, doi:10.1021/nl400258t (2013).
